# Musical Experience and the Aging Auditory System: Implications for Cognitive Abilities and Hearing Speech in Noise

**DOI:** 10.1371/journal.pone.0018082

**Published:** 2011-05-11

**Authors:** Alexandra Parbery-Clark, Dana L. Strait, Samira Anderson, Emily Hittner, Nina Kraus

**Affiliations:** 1 Auditory Neuroscience Laboratory, Northwestern University, Evanston, Illinois, United States of America; 2 Communication Sciences, Northwestern University, Evanston, Illinois, United States of America; 3 Institute for Neuroscience, Northwestern University, Evanston, Illinois, United States of America; 4 Departments of Neurobiology and Physiology, Northwestern University, Evanston, Illinois, United States of America; 5 Otolaryngology, Northwestern University, Evanston, Illinois, United States of America; Semmelweis University, Hungary

## Abstract

Much of our daily communication occurs in the presence of background noise, compromising our ability to hear. While understanding speech in noise is a challenge for everyone, it becomes increasingly difficult as we age. Although aging is generally accompanied by hearing loss, this perceptual decline cannot fully account for the difficulties experienced by older adults for hearing in noise. Decreased cognitive skills concurrent with reduced perceptual acuity are thought to contribute to the difficulty older adults experience understanding speech in noise. Given that musical experience positively impacts speech perception in noise in young adults (ages 18–30), we asked whether musical experience benefits an older cohort of musicians (ages 45–65), potentially offsetting the age-related decline in speech-in-noise perceptual abilities and associated cognitive function (i.e., working memory). Consistent with performance in young adults, older musicians demonstrated enhanced speech-in-noise perception relative to nonmusicians along with greater auditory, but not visual, working memory capacity. By demonstrating that speech-in-noise perception and related cognitive function are enhanced in older musicians, our results imply that musical training may reduce the impact of age-related auditory decline.

## Introduction

Aging negatively affects the ability to understand speech in noise (SIN) [1]–[6]. Although hearing loss can explain some of the SIN perception difficulties experienced with aging, SIN perception difficulties cannot be wholly accounted for by hearing thresholds [7]–[8]. Declines in auditory acuity [9]–[10], temporal processing [11], memory [12], speed of information processing [13]–[14] and the ability to filter out irrelevant competing auditory input [15]–[16] also contribute to difficulties reported by older adults for hearing SIN. Listening to speech in noise requires an active interplay between cognitive (e.g., attention and memory) and perceptual processes that enable the nervous system to distinguish between a target voice and competing noise [17]–[19]. As listening conditions become harder (i.e., the background noise becomes louder), hearing becomes more effortful and increasingly dependent on the recruitment of attentional and working memory resources [20]–[22]. Therefore, individuals with heightened memory capabilities may be better able to overcome the deleterious effects of background noise on perception, aiding in the retention, rehearsal and recall of the target speech signal.

Another mechanism subserving SIN perception is the ability of the auditory system to separate rapidly occurring temporal events (i.e., temporal acuity) [23]. One means of measuring auditory temporal acuity is with a backward masking paradigm in which perceptual thresholds are determined by how loud a tone needs to be for it to be perceived when directly followed by a competing signal (i.e., a masker). Backward masking not only relates to cognitive performance [24]–[25], such as auditory working memory and attention [26], but it is also negatively affected by aging [27]–[28] and may contribute to the noted poorer speech perception in older adults [29]–[30]. These age-related declines in temporal acuity and cognitive processes alongside the growth of the older population as a consequence of increasing life expectancy mean more people will experience communication difficulties, such as problems hearing in noisy environments. Reflective of the well established experience-dependent malleability of auditory function [31]–[32], considerable effort has been expended for the development of training programs that aim to improve auditory and working memory functions in older adults as a means to reduce the negative auditory impact of aging (e.g., Listening and Communication Enhancement (LACE, Neurotone Inc., Redwood City, CA, USA) and Brain Fitness (Posit Science Corp., San Fransicso, CA)).

Musicians, who have experienced life-long musical training, demonstrate a perceptual advantage for understanding speech in noise [17], [33] that is thought to be driven by auditory-related cognitive enhancements (e.g., verbal memory and auditory attention) and heightened auditory abilities. This musician advantage for speech-in-noise perception joins other work showing that musical training enhances the development of auditory skills beyond music [26], [34]–[39] such as language [40]–[42] (see [43] for a review). These musician auditory perceptual advantages are supported by functional and structural changes seen both cortically and subcortically for the processing of sound [44]–[54] and specifically for processing speech in noise [33]. Musicians are further noted to have enhancements for auditory-specific cognitive abilities, such as auditory working memory [17], [26], [55]–[58] and auditory attention [26], which may reflect the necessary integration of auditory perceptual and cognitive skills for learning a musical instrument.

Thus far, this musician enhancement for speech-in-noise perception has only been evaluated in young adults [17], [33]. Although these data imply that musical training has the potential to limit the age-related decline of SIN abilities, this cannot be determined without testing an older cohort of musicians. To define the impact of musical training on the perceptual and cognitive skills of adults in an older cohort, we assessed auditory perceptual and auditory and visual cognitive function in normal hearing musicians and non-musicians between the ages of 45–65. We hypothesized that, like young adults, older musicians demonstrate enhanced SIN perception and that this enhancement relates to greater auditory-specific cognitive and perceptual performance.

## Methods

### Subjects

Thirty-seven subjects between the ages of 45–65 were recruited from the Chicago area and gave their written informed consent according to principles set forth by Northwestern University's Institutional Review Board. We chose this transitional age group because it allowed control of audiometric hearing thresholds and cognitive factors. All subjects had normal hearing (octave frequencies from 0.125–4 kHz bilaterally ≤20 dB HL, pure tone average ≤10 dB HL), were native English speakers, and did not report neurological or learning disorders. All subjects had IQs>100 as measured by the two-subtest Abbreviated Wechsler's Adult Scale of Intelligence [59]. To control for the increasing likelihood of cognitive decline with aging, all subjects 60 years or older were screened with the Montreal Cognitive Assessment Battery [60] and demonstrated normal cognitive function (score ≥26). All experimental protocols were reviewed and approved by Northwestern University's Institutional Review Board.

Eighteen subjects were classified as musicians, all of whom had begun musical training at or before age nine and had consistently played a musical instrument throughout their lives (see Table 1). To ensure that our musicians were still musically active, we required musicians to engage in musical activities such as practicing, teaching or performing a minimum of three times a week. Nineteen subjects were classified as non-musicians. Twelve of these non-musician subjects reported no musical experience; the other seven had fewer than three years of musical experience at any point in their lives. All subjects with some degree of musical experience rated their musical proficiency on their primary instrument on a scale from 1–10. Whereas all musicians rated themselves at an 8 or higher, the seven non-musicians with minimal musical experience rated themselves at 1.5 or lower. To ensure that any observed effects could not be accounted for by differences in physical activity levels, all subjects completed a physical activity questionnaire in which participants described the type and quantity of weekly physical activity. To account for varying types of physical activity, walking and biking were given half the reported hourly value, while running, weight training, and more vigorous activities were given a full reported hourly value. The total number of hours of physical activity per week was summed and participants were assigned a final score of 0 (less than 1 hour/week), 1 (1–2 hours/week), 2 (2–3 hours/week), 3 (3–4 hours/week), or 4 (4+ hours per week). Groups were matched on physical activity levels (F(1,36) = 1.482, p = 0.517), age (F(1,36) = 0.351, p = 0.557) overall I.Q. (F(1,36) = 2.79, p = 0.204; see results below). There were no significant group differences in hearing sensitivity for all frequencies measured (0.125–12.5 kHz, F(1,12) = 0.610, p = 0.848). See Table 2 for group means.

**Table 1:** Musicians' instrumental histories.

	Years of musical experience	Age onset, yrs	Instrument
**Musicians**			
1	54	4	Piano/cello
2	50	6	Clarinet
3	49	8	Piano/French horn
4	50	7	Piano/French horn
5	54	6	Piano/Trombone
6	45	5	Piano/Violin
7	49	6	Piano
8	54	6	Piano
9	57	5	Piano
10	59	3	Piano
11	45	6	Piano
12	50	6	Piano
13	49	4	Piano
14	47	6	Piano
15	47	7	Piano
16	43	6	Violin
17	55	6	Violin
18	42	5	Oboe
**Mean**	50	5.6	

Years of musical experience, age at which musical training began and major instruments played are indicated for all musician participants.

**Table 2:** Group characteristics.

	WASI(Standard Score)	PTA (.5–4 kHz) dB HL	Age
**Musicians**Mean (SD)	125 (6.57)	8.26 (2.84)	55 (4.24)
**NonMusicians**Mean (SD)	122 (6.32)	9.66 (3.32)	54 (6.02)

Group means (standard deviations) for IQ measures (WASI), hearing thresholds (pure tone average (PTA) of the hearing thresholds at 500, 1000, 2000, 4000 Hz) and age.

### Speech Perception in Noise

#### HINT

The Hearing in Noise Test (HINT, Bio-logic Systems Corp; Mundelein, IL) [61] is an adaptive test of speech recognition that measures speech perception ability in noise. During this test, participants repeated short and semantically and syntactically simple sentences (e.g., *she stood near the window*) presented in speech-shaped background noise. Speech stimuli consist of Bamford-Kowal-Bench [62] sentences (12 lists of 20 sentences) spoken by a male and presented in free field. Participants sat one meter from the loudspeaker from which the target sentences and the noise originated at a 0 degree azimuth. The noise presentation level was fixed at 65 dB SPL and the program adjusted perceptual difficulty by increasing or decreasing the intensity level of the target sentences until the threshold signal-to-noise ratio (SNR) was determined. Perceptual SIN thresholds were defined as the level difference (in dB) between the speech and the noise presentation levels at which 50% of sentences are correctly repeated. A lower SNR indicates better performance.

#### QuickSIN

The Quick Speech-in-Noise Test (QuickSIN, Etymotic Research; Elk Grove, IL) [63] is a non-adaptive test of speech perception in which speech is presented binaurally in four-talker babble noise (three females and one male) through insert earphones (ER-2, Etymotic Research, Elk Grove Village, IL). Four lists of sentences were administered, with each list consisting of six sentences. Sentences were presented at 70 dB SPL, with the first sentence starting at a SNR of 25 dB and each subsequent sentence presented with a 5 dB SNR reduction down to 0 dB SNR. The sentences are syntactically correct yet do not contain many semantic or contextual cues [64]. Participants were asked to repeat each sentence and their SNR loss was based on the number of target words correctly recalled. Sample sentences, with target words underlined, include, “*The squarepeg will settle in the roundhole* .” and “*The sense of smell is betterthan that of touch*.” The total number of key words correctly recalled in the list (30 in total) is subtracted from 25.5 to give the final SNR loss ((see Killion et al. 2004 and the QuickSIN User's Manual (Etymotic Research 2001) for further details)). The final score is the average SNR loss scores across the four lists. A lower SNR loss value is indicative of better performance.

#### WIN

The Words in Noise Test (WIN) [65] is a non-adaptive test of speech perception in four-talker babble noise (three females and one male), presented binaurally to participants through Etymotic ER-2 insert earphones [65]. Participants were asked to repeat the words they heard after the carrier phrase, “Say the word ________”. Thirty-five words were presented with a starting dB SNR of 24, decreasing in 4 dB steps until 0 dB; five words are presented at each SNR level. The final SNR score was based on the number of correctly repeated words. A lower score indicates better performance.

### Working Memory

#### Auditory

The Woodcock-Johnson III Test of Cognitive Abilities [66] subtests for auditory working memory (AWM) and memory for numbers reversed (NR) were used to assess working memory, both of which required participants to store and reorder aurally-presented information. For AWM, participants reordered a dictated series of digits and nouns by first repeating the nouns in sequential order and then repeating the digits in sequential order (e.g., the correct ordering of the following sequence, “4, salt, fox, 7, stove, 2, 9, boot” is “*salt, fox, stove, boot*” and “*4, 7, 2, 9*”). For NR, participants repeated a sequence of numbers in reverse order. The most difficult item contained eight digits (e.g., “9, 6, 1, 3, 7, 4, 5, 2” which in reverse would be “*2, 5, 4, 7, 3, 1, 6, 9*”). A working memory cluster score was computed based on scores from the AWM and NR subtests. Age-normed standard scores were used for all statistical analyses. A higher score indicates better performance.

#### Visual

The Colorado Assessment Test's Visual Working Memory subtest (VWM) [67] is an adaptive test for which participants are instructed to monitor a screen containing eight blue boxes that change color one at a time. The first trial begins with two boxes sequentially changing color. Participants were asked to click on the boxes in the order they changed color. The number of boxes changing color increases with successive correct replies. Participants completed both forward and reversed conditions. The final VWM score was an average of the participant's performance on both forward and reversed conditions. A higher score indicates better performance.

### Auditory Temporal Acuity: Backward Masking

The backward masking subtest from the IHR Multi-centre Battery for Auditory Processing was employed to assess backward masking acuity (Medical Research Council Institute of Hearing Research, Nottingham, UK) [68]. The subtest employed a three-alternative forced choice paradigm in the form of an animated computer game. Three characters opened their mouths to “speak” masking noise sounds (band-pass noise with a center frequency of 100 Hz, a width of 800 Hz and duration of 300 ms). A 90 dB target tone was presented before one of the three noise sounds, with the tone offset coinciding with the noise's onset. The target tone was equally distributed between the three characters. Participants indicated which character was the “odd-one-out” (i.e., which character presented the target tone prior to the masker, rather than the masker alone) by pressing the corresponding button on a response box. The target tone presentation level was then increased or decreased depending on the participant's performance (correct responses → decrease in dB; incorrect responses → increase in dB). An adaptive staircase method was employed (3 down, 1 up), yielding a minimum detectable threshold level in dB (see Amitay et al, 2006 [69] for further description). A lower threshold indicates better task performance (i.e., the target tone is perceived at quiet levels).

### Data Analysis

Data were analyzed using a multivariate analysis of variance (MANOVA). All results reflect two-tailed values. Normality for all data was confirmed by the Komogorov Smirnov test for equality. Relationships between SIN perception, cognitive function and auditory acuity were explored with Pearson's correlation analyses. Statistical analyses were conducted with SPSS (SPSS Inc., Chicago, IL).

## Results

Musicians demonstrated greater proficiency on perceptual and auditory-based cognitive measures than non-musicians. Specifically, musicians had enhanced speech-in-noise perception, auditory working memory and auditory temporal acuity (lower backward masking thresholds), compared to non-musicians.

**Figure 1:**
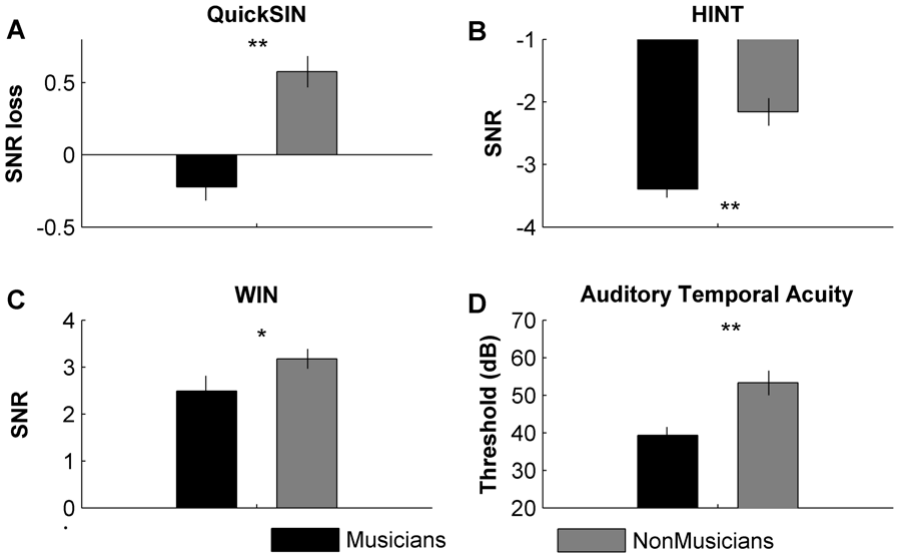
Performance for musicians and non-musicians on speech-in-noise and backward masking tasks. Musicians demonstrated enhanced performance for all three measures of speech-in-noise perception (QuickSIN, HINT and WIN), indicating that they were better able to hear in more challenging signal-to-noise ratios (SNRs). Musicians performed better (i.e., had lower thresholds) on the auditory temporal acuity test as assessed by backward masking. Error bars represent one standard error. * p<0.05 ** p<0.01.

**Figure 2:**
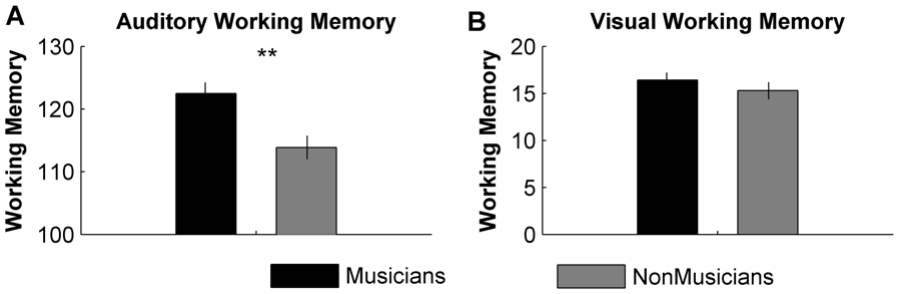
Performance for musicians and non-musicians on working memory tasks. Musicians demonstrated significantly better auditory working memory than non-musicians, but no enhancement for visual working memory. Error bars represent one standard error. ** p<0.01.

Musicians demonstrated lower thresholds than non-musicians for all three speech-in-noise tests (Fig. 1; HINT: F(1, 36) = 22.49, p<0.005); QuickSIN: F(1, 36) = 33.11, p<0.005); WIN: F(1,36) = 4.709, p = 0.04), better performance on auditory working memory composite (AWM: F(1, 36) = 16.34, p<0.005) and higher auditory temporal acuity (i.e., lower backward masking thresholds) (Fig. 1; (F(1,36) = 13.47, p = 0.001). Visual working memory (VWM) scores did not differ between the groups (Fig. 2; F(1,36) = 1.148, p = 0.291; see Table 3 for group means and standard deviations).

**Table 3:** Group behavioural performance.

	HINTSNR	QuickSINSNR loss	WINSNR	BM (dB)	AWM	VWM
**Musicians**	−3.37 (0.52)	−0.22 (0.39)	2.48 (1.37)	39.35 (9.23)	124 (9.19)	16.38 (3.18)
**NonMusicians**	−2.24 (0.87)	0.51 (0.38)	3.3 (0.85)	53 (13.28)	110 (11.18)	15.21 (3.48)
***group comparison*** ***p - value***	<0.005	<0 .005	<0.04	<0.001	<0.005	>0.2

Group means (standard deviations) for the speech-in-noise tests (HINT, QuickSIN and WIN), backward masking (BM), auditory working memory, (AWM) and visual working memory (VWM). For all auditory tests musicians outperformed the non-musicians (backward masking and auditory working memory), however, group performance was equivalent for the visual working memory.

**Figure 3:**
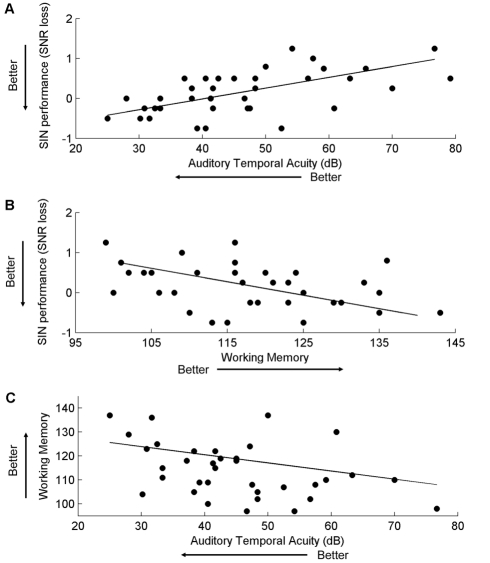
Correlations between measures of speech-in-noise perception, working memory and auditory temporal acuity. Better performance on QuickSIN (more negative) was correlated with lower (better) auditory temporal acuity as assessed by backward masking thresholds (top: r = 0.573, p<0.005) and higher working memory ability (middle: r = −0.402, p = 0.014). Working memory and backward masking thresholds were also correlated (bottom: r = −0.495, p = 0.002).

Auditory working memory ability correlated with SIN perception, with better AWM performance relating to better performance on QuickSIN (r = −0.402, p = 0.014) and HINT (r = −0.351, p = 0.033) but not WIN (r = −0.169, p = 0.316). Backward masking performance correlated with all SIN tests, with lower (better) backward masking thresholds corresponding to the ability to understand speech in noise at lower SNRs (QuickSIN: r = 0.573, p<0.005; HINT: r = 0.411, p = 0.012; WIN: r = 0.372, p = 0.023). A relationship between backward masking thresholds and auditory working memory was also observed (Fig. 3; r = −0.495, p = 0.002). To ensure that the observed correlations between auditory working memory and SIN performance (QuickSIN and HINT) were not an artifact of musicians' enhanced auditory working memory, we defined the relationships between these variables for the musician and non-musician group through separate analyses. Within-group correlations were absent for these measures (see Tables S1), indicating that the relationships between auditory working memory and SIN performance are present only when the two groups are combined. This suggests that these cognitive-perceptual relationships are not driven by the musician group's enhanced auditory working memory.

Speech in noise performance as measured by the QuickSIN related with speech in noise performance as measured by the HINT (r = 0.510, p = 0.001) and the WIN (r = 0.329, p = 0.047). No significant relationship, however, was observed between HINT and WIN performance (r = 0.199, p = 0.236), suggesting that performance on these two tests may rely on different mechanisms. With regards to IQ, no group differences were found for overall IQ (F(1,36) = 2.79, p = 0.204) or for the Matrix Reasoning subtest (WASImr: F(1,36) = 6.979, p = 0.271). Musicians did, however, demonstrate higher performance on the Vocabulary subtest (WASIv: F(1,36) = 6.979, p = 0.012). Still, the reported musician advantages for SIN perception, auditory working memory and temporal resolution were not driven by WASIv performance (see Results S1 and Table S2 for further details).

### Assessing relationships between years of musical experience, age of onset and perceptual and cognitive performance

To investigate the relationships between musical experience and the perceptual (SIN perception, temporal resolution) and cognitive measures (working memory and WASI), correlational analyses were employed. Within the musician group only, age of onset of musical training did not relate to the perceptual or cognitive measures (see Table 4). However, we only have a limited range of data points (6 years) for this inclusionary measure, as musicians were required to have started musical training before the age of 8. Therefore, the lack of correlation between age of musical training onset and the perceptual and cognitive skills likely speaks to the restricted range of age of onset rather than being a true indicator of a lack of relationship between these variables. Similarly, years of musical experience did not relate to the perceptual or cognitive measures (see Table 5). However, it is important to critically evaluate the nature of these variables before concluding that no relationship exists between them. Perceptual and cognitive skills do not improve monotonically over the lifespan; in fact, these skills are negatively affected by age [1]–[6], [27]–[28], and the older adults tested here are likely to be affected by age-related decline. Accordingly, we did not predict significant correlations between the linear increase in years of musical experience and these nonlinear perceptual and cognitive measures. In summary, while correlational analysis has proven useful for quantifying the extent of musical practice in children and young adults, its application to this older population is inherently misleading.

**Table 4:** Relationship between age of onset and years of practice with perceptual and cognitive measures (musicians only).

Musicians Only		WASI	Auditory Working Memory	Visual Working Memory	QuickSIN	WIN	HINT	Temporal Resolution
Age of Onset	rho	−0.267	−0.007	0.271	0.091	−0.194	0.124	0.090
	p value	0.284	0.979	0.277	0.719	0.440	0.624	0.723

Correlations between age of onset and the cognitive and perceptual measures for the musician group only. No significant relationships were found between age of onset and these cognitive and perceptual measures. In this study, age of onset was an inclusionary measure (musicians were required to have started musical training before the age of 8 years) resulting in a restricted range of data points (6 years). Therefore, the lack of correlation between age of onset and the perceptual and cognitive skills likely speaks to the limited range of age of onset rather than being a true indicator of a lack of relationship between these variables.

**Table 5:** Relationship between years of practice with perceptual and cognitive measures (musicians only).

Musicians Only		WASI	Auditory Working Memory	Visual Working Memory	QuickSIN	WIN	HINT	Temporal Resolution
Years of experience	rho	−0.047	−0.139	−0.093	0.322	−0.211	−0.086	−0.033
	p value	0.853	0.581	0.715	0.193	0.379	0.735	0.101

Correlations between years of musical experience and the cognitive and perceptual measures for the musician group only. No significant relationships were found between years of musical experience and these cognitive and perceptual measures. However, perceptual and cognitive skills do not improve monotonically over the lifespan, rather they are negatively affected by age. The absence of a significant relationship is not surprising given the linear nature of years of musical experience and these nonlinear perceptual and cognitive measures.

## Discussion

We herein demonstrate enhanced speech-in-noise perception for older adult musicians between the ages of 45–65 which correlates with auditory cognitive and perceptual performance. As with young adult musicians, older adult musicians demonstrate increased auditory working memory capacity and increased auditory temporal acuity (as measured by backward masking), which may undergird the perception of speech in noise. As such, our results indicate that musical training may serve as a means to offset the effects of age-related communication disorders by improving hearing in noise – an everyday listening skill – through the enhancement of auditory-related perceptual and cognitive functions.

### Auditory working memory contributes to speech-in-noise perception

Auditory working memory is an important component of language comprehension, even in the absence of background noise [70]–[72]. The addition of background noise reduces one's auditory working memory capacity [73]–[74], resulting in the decreased ability to rehearse and recall a target speaker's utterance [22], further compromising the perception of a speech signal already degraded by noise. We recently demonstrated improved auditory working memory capacity and SIN perception in young adult musicians as well as a link between performance on both tasks [17], adding to a growing body of research indicating the importance of auditory-related cognitive abilities for SIN perception [21]. In the present study, older musicians also demonstrate enhanced auditory working memory and SIN perception. This suggests that lifelong musical training may confer advantages for an older population in two everyday human functions that are known to decline with age.

A number of studies have evidenced a musician enhancement for auditory working and verbal memory [17], [55]–[56], [58], [75]–[76]. While some research has reported musician enhancements for only auditory and not visual working memory [55], [58], others have found enhancements for both auditory and visual memory [57]. Further complicating matters, it appears that musical training may have distinct effects on working memory abilities at different stages of development, with musically trained children demonstrating superior verbal and non-verbal working memory but musically trained adults demonstrating only superior verbal working memory [77]. While there has been some debate over this work (i.e., musician groups having higher IQ, see Schellenberg & Peretz 2007, Schellenberg, 2006; 2008; 2009 for a review [78]–[81]), neural evidence suggests that musicians employ different brain structures for auditory memory tasks, thus providing a neural correlate of improved memory in musicians [82]–[84]. Here we demonstrate that older musicians have greater auditory working memory capacity, which may contribute to their improved SIN perception. Additionally, our results indicate a musician enhancement for auditory, but not visual, working memory, supporting the notion that life-long musical training refines skills most relevant to musical processing, namely auditory skills, rather than improving memory in a domain-general fashion.

### Auditory temporal acuity relates to speech-in-noise perception

Auditory temporal acuity, as measured by backward masking performance, has been linked to speech perception abilities [30] and its decline with age, even in normal hearing older adults [27]–[28], is thought to contribute to the commonly reported speech perception difficulties in this population. Consistent with results reported in young adults, the present data indicate that long-term musical experience shapes speech-in-noise perception [17] and auditory temporal acuity, as assessed by backward masking perception [26]. Although the brain mechanisms underlying these perceptual enhancements remain undetermined, there is growing evidence that musical training hones auditory perception through the neural tuning of auditory pathway mechanisms (reviewed in Kraus & Chandrasekaran, 2010 [43]). Auditory perceptual learning is thought to be driven in a top-down manner, with cortical functions refining neural encoding at earlier stages in the processing stream, leading to increased perceptual performance [85]–[88]. The refinement of lower level auditory structures via top-down control is thought to lead to the neural encoding of signals at higher internal SNRs, which in turn contribute to heightened auditory perception through a more efficient auditory system [26], [43], [89]–[90]. As such, this top-down mechanism provides a possible explanation for the musicians' improved performance on backward masking tasks and SIN perception. In light of increasing problems with auditory processing experienced by older adults [91]–[92], our results indicate that lifelong musical training might limit the degradative effects of aging.

### Conclusion and future directions

The demographic shift towards an increasingly older population is accompanied by an increase in the prevalence of perceptual and cognitive disorders. One means of offsetting or slowing down age-related declines may be through engaging in mentally stimulating activities [93], such as musical practice [94]. While research into the impact of musical training on aging processes is a new avenue of investigation, our results indicate a positive role of lifelong musical training on auditory perception and cognitive processes. It is also possible that musical training during developmental years enhances working memory, temporal resolution and SIN and that these effects are carried forward throughout the lifespan. Additional research might tease apart these two possibilities by comparing cognitive and perceptual performance in older adults who ceased musical training at different developmental stages with those who have engaged in musical activities throughout their lives. Regardless of the outcome, the results presented here indicate that older adults with extensive musical backgrounds are better equipped to deal with the auditory perceptual demands of real-world situations. Although more work is needed to determine the efficacy of using music as a management strategy for perceptual and cognitive declines, these results underscore the potential remediatory benefits of musical training for an aging population.

## Supporting Information

Results S1 (DOC)

Table S1 To verify that the observed correlations between auditory working memory and SIN performance (QuickSIN and HINT) were not an artifact of musicians' enhanced auditory working memory, the relationships between these variables for the musician and non-musician group through separate analyses were explored with correlational analyses. Within-group correlations were absent for these measures.
(DOCX)

Table S2 The musician advantage for auditory working memory, temporal resolution and SIN perception remains even when covarying for WASI vocabulary.
(DOCX)
