# Supplementary material for: Musical Experience and the Aging Auditory System: Implications for Cognitive Abilities and Hearing Speech in Noise
Source: PLoS One. 2011 May 11;6(5):e18082. doi: 10.1371/journal.pone.0018082 (PMC3092743; doi:10.1371/journal.pone.0018082)
Supplement: Results S1 — (DOC) [file pone.0018082.s001.doc]

**Supplemental Results**

No group differences were observed for overall IQ (F(1,36) = 2.79, p= 0.204) or for the Matrix Reasoning subtest (WASImr: F(1,36) = 6.979, p = 0.271). Musicians did, however, exhibit higher performance on the Vocabulary subtest (WASIv: F(1,36) = 6.979, p = 0.012). WASIv also correlated with temporal resolution (r = -0.448, p = 0.005), QuickSIN (r = -0.324, p = 0.033), HINT (r = -0.352., p = 0.05) and auditory working memory (r = 0.561, p = 0.001). Still, the musician advantage on the reported cognitive and perceptual measures (i.e., temporal resolution, auditory working memory and SIN perception) could not be accounted for by WASIv group differences as the musicians outperformed the nonmusicians on these measures even when covarying for the WASIv subtest (ANCOVA) (see Table S2). WASImr did not correlate with the cognitive or perceptual tests (all r values > 0.1, all p values > 0.2).
