# Supplementary material for: Musical Experience and the Aging Auditory System: Implications for Cognitive Abilities and Hearing Speech in Noise
Source: PLoS One. 2011 May 11;6(5):e18082. doi: 10.1371/journal.pone.0018082 (PMC3092743; doi:10.1371/journal.pone.0018082)
Supplement: Table S1 — To verify that the observed correlations between auditory working memory and SIN performance (QuickSIN and HINT) were not an artifact of musicians' enhanced auditory working memory, the relationships between these variables for the musician and non-musician group through separate analyses were explored with correlational analyses. Within-group correlations were absent for these measures. (DOCX) [file pone.0018082.s002.docx]

**Table S1: Relationships between QuickSIN, HINT and auditory working memory in the musician and non-musician group separately.**

|  | Musicians only | | Non-musicians only | |
| --- | --- | --- | --- | --- |
|  | QuickSIN | HINT | QuickSIN | HINT |
| Auditory working memory | r = 0.075 | r = -0.290 | r = -0.066 | r = 0.196 |
|  | p = 0.769 | p = 0.243 | p = 0.788 | p = 0.421 |
