# Supplementary material for: Musical Experience and the Aging Auditory System: Implications for Cognitive Abilities and Hearing Speech in Noise
Source: PLoS One. 2011 May 11;6(5):e18082. doi: 10.1371/journal.pone.0018082 (PMC3092743; doi:10.1371/journal.pone.0018082)
Supplement: Table S2 — The musician advantage for auditory working memory, temporal resolution and SIN perception remains even when covarying for WASI vocabulary. (DOCX) [file pone.0018082.s003.docx]

**Table S2: Group differences on the perceptual and cognitive measures with and without covarying for WASI vocabulary.**

|  | Original Analysis | Co-varying for WASI Vocabulary |
| --- | --- | --- |
| Auditory Working Memory | F = 16.34 | F = 8.33 |
|  | p < 0.005 | p = 0.007 |
| Temporal Resolution | F = 13.47 | F = 7.373 |
|  | p = 0.001 | p = 0.01 |
| HINT | F = 22.49 | F = 16.46 |
|  | p < 0.005 | p < 0.005 |
| QuickSIN | F = 33.11 | F = 24.603 |
|  | p < 0.005 | p < 0.005 |
| WIN | F = 4.709 | F = 3.715 |
|  | p = 0.042 | p = 0.062 |
| Visual Working Memory | F = 1.148 | F = 0.48 |
|  | p = 0.291 | p = 0.828 |
